# Supplementary material for: Effectiveness of an Intervention of Dietary Counseling for Overweight and Obese Pregnant Women in the Consumption of Sugars and Energy
Source: Nutrients. 2019 Feb 13;11(2):385. doi: 10.3390/nu11020385 (PMC6412784; doi:10.3390/nu11020385)
Supplement: Supplementary file 1 [file nutrients-11-00385-s001.pdf]

Table S1. Effect of the nutritional intervention on the change of consumption of total sugars and energy. Linear regression models.

	MODEL 1	*P*-Value	MODEL 2	*P*-Value	MODEL 3	*P*-Value
Changes in total sugars consumption, g/day	−19.64 (−32.60, −6.67)	0.003	−15.44 (−25.04, −5.84)	0.002	−15.23 (−25.01, −5.46)	0.002
Change in total kilocalorie consumption, kcal/day	−210 (−352, −69)	0.004	−126 (−239, −14)	0.028	−125 (−239, −10)	0.032
Change in total percentage of energy consumption derived from sugars, %	−1 (−3, 1)	0.060	−2 (−3, −1)	0.001	−2 (−3, −1)	0.002

Reference group is the control group. Figures represent β coefficients of the regression models and 95% confidence intervals. MODEL 1: Change in sugars/kilocalories/% calories from sugars at 35–37 weeks of pregnancy= β0 + β1 (dietary counseling). MODEL 2: Change in sugars/kilocalories/% calories from sugars at 35–37 weeks of pregnancy = β0 + β1 (dietary counseling) + β2 (sugars/kilocalories/% calories at <15 weeks of pregnancy). MODEL 3: Change in sugars/kilocalories/% calories from sugars at 35-37 weeks of pregnancy = β0 + β1 (dietary counseling) + β2 (sugars/kilocalories/% calories at <15 weeks of pregnancy) + β3 (marital state) + β4 (persons in home) + β5 (age) + β6 (level of studies) + β7 (monthly income) + β8 (relation to head of household) + β9 (activity) + β10 (depression) + β11 (nutritional status) + β12 (gestational weight gain) + β13 (season) + β14 (health center).
